# Comparison between Acupuncture and Nicotine Replacement Therapies for Smoking Cessation Based on Randomized Controlled Trials: A Systematic Review and Bayesian Network Meta-Analysis

**DOI:** 10.1155/2021/9997516

**Published:** 2021-06-16

**Authors:** Runjing Dai, Yongchun Cao, Hailiang Zhang, Na Zhao, Dong Ren, Xiaomei Jiang, Guisen Zheng, Shisan Bao, Xingke Yan, Jingchun Fan

**Affiliations:** ^1^School of Public Health, Center for Evidence-Based Medicine, Gansu University of Chinese Medicine, Lanzhou, Gansu 730000, China; ^2^Human Resources Management Department, Gansu Provincial Maternity and Child-Care Hospital, Lanzhou, Gansu 730050, China; ^3^Psychosomatic and Sleep Medicine, Gansu Gem Flower Hospital, Lanzhou, Gansu 730060, China; ^4^College of Acupuncture-Moxibustion and Tuina, Gansu University of Chinese Medicine, Lanzhou, Gsnsu 730000, China

## Abstract

**Objectives:**

To evaluate the efficacy and/or safety of acupuncture therapy (AT) in quitting smoking.

**Methods:**

Randomized controlled trials (RCTs) were searched in PubMed, Cochrane Library, Embase, Web of Science, and Chinese Biomedical Database (CBM). We used Cochrane Collaborative Quality Assessment to assess the risk of bias. Bayesian network meta-analysis was utilized to evaluate the efficacy and safety of different interventions. Data analyses were conducted using WinBUGS 1.4.3, Stata 14, and RevMan 5.3.5 software.

**Results:**

A total of 2706 patients from 23 studies were included, involving 6 treatment arms. Network meta-analysis demonstrated that there was no significant difference in short-term abstinence rates or changes in Fagerstrom test for nicotine dependence (FTND) scores and daily smoking among these groups (AT, sham acupuncture therapy (SAT), auricular acupressure (AA), sham auricular acupressure (SAA), acupuncture plus auricular acupressure (APAA), and nicotine replacement therapy (NRT)). However, there was a significant difference between SAA and AA with risk ratio (RR) of 2.49 (95% CI 1.14, 5.97) in long-term abstinence rate. The probabilistic ranking results showed that APAA and AA were superior to other interventions in the comparison of abstinence rates. There was no obvious inconsistency between the direct comparison and indirect comparison, using the consistency test.

**Conclusion:**

AA was superior to SAA in smoke quitting, but there was no difference among other interventions in long-term truncation rates. There was no difference in short-term abstinence rates among these selected groups. We need large sample RCTs to clarify the advantages of interventions such as APAA and AA. In addition, reporting of adverse events that may occur during treatment also should be enhanced to complement evidence-based medicine. The trial is registered with PROSPERO CRD42020164712.

## 1. Introduction

Tobacco dependence is one of the major public health problems [1], which is closely related to the development of lung cancers and cardiovascular diseases [2]. The information from WHO illustrates that mortality is >7 million or ∼1.2 million people worldwide from direct tobacco or second-hand smoke [3]. Smoking not only contributes to the development of these chronic diseases but also is a huge economic burden [1]. Smoking, however, is a preventable cause of leading death and disability by smoking cessation to reduce the risk of illness and early death [4–6]. Therefore, quitting smoking is a great benefit from health and economic burden points of view [1].

The most internationally recognized standard therapy is nicotine replacement with the objective quantification of abstinence rate [7, 8]. However, there are a number of alternative approaches that have been tested for quitting smoke, including acupuncture, auricular acupressure, acupuncture plus auricular acupressure (APAA), and other traditional Chinese medicines [9–11]. It has been suggested that acupuncture and auricular acupressure (a traditional Chinese medicine) are effective on smoking cessation [12, 13], but their efficacy is still controversial. Meta-analysis on smoking cessation of acupuncture and auricular acupressure is different, which might be due to overlapping and redundancy of topics [8, 14]. El Bahri et al. extracted randomized and quasirandomized controlled trials (quasi-RCTs) to evaluate the effect of acupuncture and its derivatives on smoking cessation, showing that the effect of acupuncture on smoking cessation is affected by variation [15]. The advantages of nicotine replacement therapies are convenient with effect immediately, whereas the advantage of acupuncture therapy is diverse types, low price, and easier to be accepted by the public. On the other hand, there are some disadvantages for either nicotine replacement therapies or acupuncture therapy, such as dizziness, headache, or producing allergic phenomena in patients. Moreover, the efficacy of smoking quitting by acupuncture and other interventions is evaluated based on direct comparison, but the safety comparisons of different treatments are limited. Thus, in the current study, we aim to evaluate whether nicotine replacement therapies or acupuncture therapy is better than others, which could offer some evidence for the readers to understand the importance of this information.

## 2. Methods

### 2.1. Protocol and Registration

Our study has been registered on the International Prospective Register of Systematic Reviews (PROSPERO) and the registered number is CRD42020164712 which is available at https://www.crd.york.ac.uk/PROSPERO/.

### 2.2. Data Sources and Search Strategy

Five electronic databases were searched from inception to March 2021 for RCTs: PubMed, the Cochrane Library, Embase, Web of Science, and Chinese Biomedical Database (CBM). Forward and reverse citation screening through a systematic review of citation and bibliography were also conducted. We used the following search terms: smoking cessation, nicotine replacement therapy, acupuncture, and auricular acupressure, as well as synonyms and derivatives of these words. There are no language restrictions. Search strategies were demonstrated for searching the English and the Chinese e-libraries (Supplementary Table 1).

### 2.3. Eligibility Criteria

We included RCTs with acupuncture therapy (AT), auricular acupressure (AA), and acupuncture combined with auricular acupressure (APAA) as intervention measures for smokers who voluntarily quit smoking. The controls included in the study were sham acupuncture therapy (SAT), sham auricular acupressure (SAA), nicotine replacement therapy (NRT), or AA alone. The literature published in Chinese and/or English languages was included. To minimize heterogeneity, the smokers who received two or more treatment combinations (except auricular acupoint combination acupuncture) or any acupuncture with electrical or laser stimulation were excluded. Furthermore, articles with duplicate data, incomplete data, or no access to the full text were excluded.

### 2.4. Study Selection and Data Extraction

We imported the retrieved literature into Endnote X9. Two independent reviewers screened and included studies according to the eligibility criteria. For any disagreements between these two reviewers, a third senior researcher was consulted, and a conclusion was reached after the discussion. For articles with duplicate data, we only included one with a larger sample size or a longer follow-up period.

We used a predesigned Excel sheet to extract data from included studies. The main information includes the first author, year of publication, sample size, interventions, methods, and outcomes. When data were reported for multiple time periods, data at each time period were recorded.

### 2.5. Outcome Measures

We selected short-term and long-term abstinence rates as primary outcome measures. We preferred the sustained abstinence rates, and if the article did not report the results of sustained abstinence outcome, we chose the point abstinence rate. As stated above, the golden standard of abstinence rate is breath carbon monoxide, urinary cotinine, or both. We have adopted the principle of this standard in the current network meta-analysis accordingly [8]. Moreover, we chose the change of the scores of Fagerstrom test for nicotine dependence (FTND) and daily smoking prior to and after treatment as the secondary outcome measure.

### 2.6. Risk of Bias Assessment

Using the Cochrane Collaborative Quality Assessment tool, two independent reviewers evaluated the methodology quality of each included study [16]. Assessment tools included random sequence generation, allocation concealment, blinding of participants and personnel, blinding of outcome assessment, incomplete outcome data, selective reporting, and other biases. For each study, the assessment criteria were defined in three levels of low risk, high risk, and unclear risk. Any disagreement between the reviewers was resolved through discussion with a third senior reviewer.

### 2.7. Date Synthesis and Analysis

Our study combined direct and indirect evidence from all available RCTs. Bayesian network meta-analysis was performed, using WinBUGS (V.1.4.3; MRC Biostatistics Unit, Cambridge University, UK) software, and Stata (V.14; StataCorp) was used to draw the network diagram, while the Review Manager Software (V.5.3.5; RevMan) was used to produce funnel plot to assess the risk of publication bias. We applied visual evaluation to evaluate the small size effect of test funnel plot asymmetry.

To measure the effects, weighted mean difference (WMD) was calculated for continuous data with 95% CI (confidence interval); risk ratio (RR) for short-term and long-term abstinence rates with 95% CI was calculated. We carried out a consistency model analysis on the main efficacy indicators. We calculated the probability of the lower surface of the cumulative sequencing curve (SUCRA) [17], ranked the optimal treatment measures, and performed node model analysis on the closed-loop network diagram to evaluate the consistency. Statistical heterogeneity [18, 19] was assessed by *χ*^2^ statistics and *I*^2^ statistics. When the *P* value was ≤0.05 and *I*^2^ was ≥50%, statistical heterogeneity existed, and the random effect model was adopted. The fitting degree of the model was judged by comparing the Deviance Information Criterion (DIC) values of the random effect model and the fixed effect model. When the difference value of DIC < 5, the fitting degree of the two models is consistent, and if DIC > 5, the model with a smaller DIC value is adopted. The model convergence was evaluated by the Potential Scale Reduction Factors (PSRF), and the closer the PSRF value was to 1, the better the convergence of the model was. Finally, two independent reviewers evaluated the quality of direct and indirect evidence using the recommended rating, development, and rating methods [20].

## 3. Results

### 3.1. Study Search

**Figure 1:**
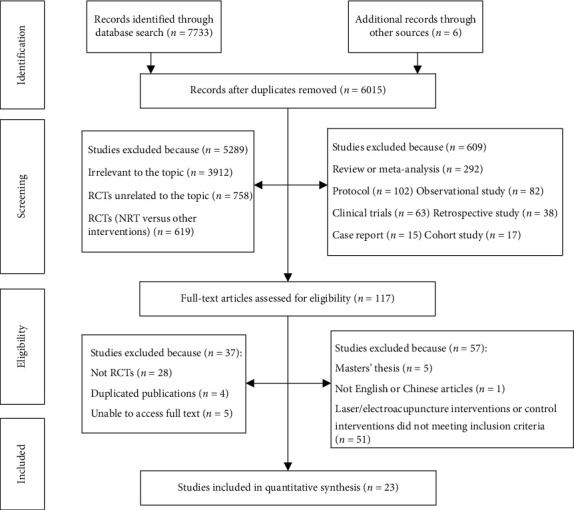
Flow diagram depicting the study selection.

A total of 7,733 references were identified from the five electronic databases with 6 records from other sources. We retrieved 117 articles for further evaluation after screening them by title and abstract. Furthermore, 94 articles were excluded for the following reasons: unable to access full text (*n* = 5), not RCTs (*n* = 28), masters' thesis (*n* = 5), not English or Chinese articles (*n* = 1), duplicate publications (*n* = 4), and laser/electroacupuncture interventions or control interventions not meeting inclusion criteria (*n* = 51). Finally, 23 RCTs [11, 13, 21–41] were included with 2706 patients. The details are shown in Figure 1.

### 3.2. Study Characteristic and Quality Assessment

**Figure 2:**
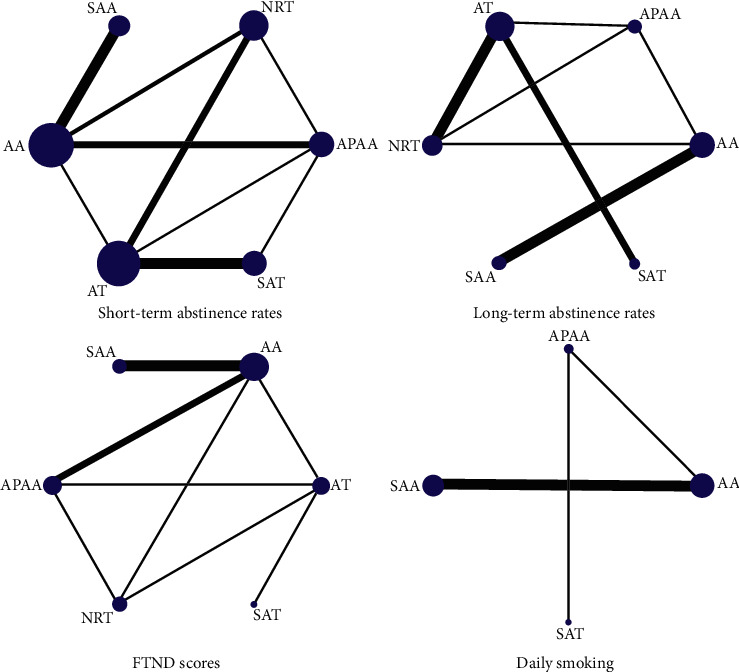
Network maps of short-term abstinence rates, long-term abstinence rates, FTND scores, and daily smoking.

Table 1 showed the characteristics of the 23 RCTs that met the criteria for our network meta-analysis. The studies have been published between 1978 and 2019, consisting of a total of 2706 patients from these studies and 6 treatment arms. The treatment period was between 10 days and 8 weeks, and the follow-up time was as short as 2 weeks and as long as 4 years. Among the 23 RCTs, 18 [11, 21, 23–32, 36–41] reported short-term abstinence rates, 8 [11, 21, 24, 30, 31, 36, 38, 41] reported long-term abstinence rates, 6 [11, 13, 21, 22, 29, 36] reported FTND scores, and 7 [13, 22, 25–27, 29, 41] reported daily smoking.  Figure 2 graphically displays the networks of evidence for all outcomes.

All the studies mentioned “randomized,” but only 39.1% (9/23) [11, 13, 22, 26, 29, 33–36] of them were truly random, using random number tables, lotteries, or computer randomization and were judged as low risk of bias. There were three (3/23) [11, 26, 34] studies that mentioned allocation concealment and nine (9/23) [13, 22, 26, 27, 31, 33–35, 39] that mentioned implementation of participants and personnel blindness. Few studies have mentioned blinding of outcome assessment, three (3/23) [22, 26, 34] trials judged “low,” and one (1/23) [25] trial judged “high.” We considered that 18 studies (18/23) were at low risk of biases with incomplete outcome data. It was judged that all the studies were of low risk in selective reporting, according to the consistent evaluation between the main results of the study and the actual results reported. The overall quality of the evidence for all the results was low, because most of the studies are lacking details. The risk assessment of bias in included studies is shown (Supplementary Figures 1 and 2).

### 3.3. Heterogeneity Analysis

We analyzed the consistency of the four outcome measures separately. The results show that the PSRF is 1, which indicates good data convergence. The heterogeneity test results showed that the heterogeneity was small in the short-term and long-term abstinence rates and daily smoking without significant difference. The random effect model was chosen because there was greater heterogeneity in the FTND score (Supplementary Table 2).

### 3.4. Model Fitting Degree Check

The results showed that the DIC values of short-term abstinence rate fixed effect model and random effect model were 97.3 and 70.36, the DIC values of long-term abstinence rate fixed effect model and random effect model were 97.27 and 29.38, the FTND scores of fixed effect model and random effect model were 34.23 and 27.69, and the DIC values of fixed effect model and random effect model of daily smoking amount were 34.23 and 28.34, respectively. The difference between the two models in each indicator is >5, so the model with a smaller DIC value was selected (random effect model). We tested the model fitting degree of each outcome index under the random effect model, and the ratio value was all close to 1, which proved that the model fitting degree was good.

### 3.5. Network Meta-Analyses

#### 3.5.1. Short-Term Abstinence Rates

**Figure 3:**
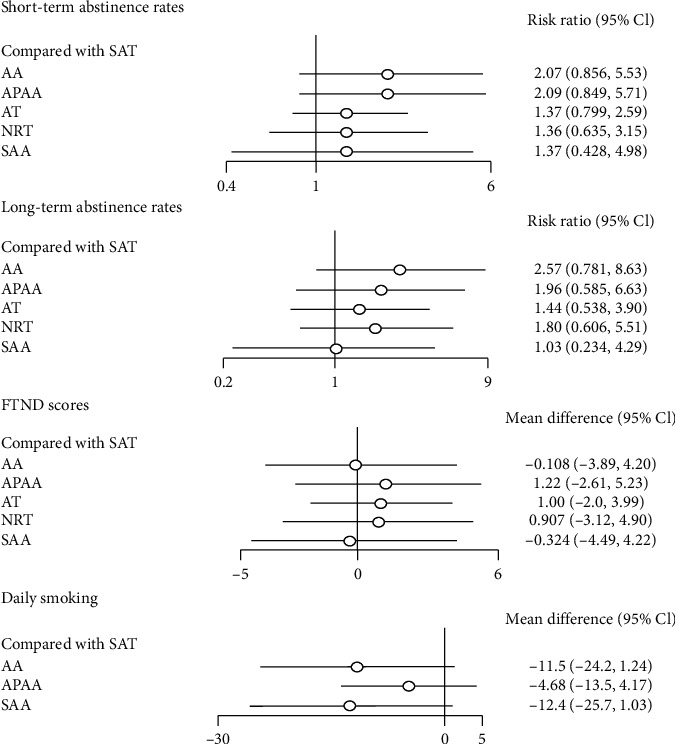
Direct pairwise random effect meta-analyses of outcomes.

We collected 18 RCTs on short-term abstinence rates. Direct pairwise randomized effect meta-analysis illustrated that there was no significant difference when AA, APAA, AT, NRT, or SAA was compared to placebo SAT, showing RR and 95% CI was 2.07 (95% CI 0.856, 5.53), 2.09 (95% CI 0.849, 5.71), 1.37 (95% CI 0.799, 2.59), 1.36 (95% CI 0.635, 3.15), or 1.37 (95% CI 0.428, 4.98), respectively (Figure 3). Network meta-analysis showed that there was no significant difference in short-term abstinence rates of 1.01 (95% CI 0.54, 1.90), 0.66 (95% CI 0.31, 1.42), 0.65 (95% CI 0.33, 1.30), and 0.66 (95% CI 0.30, 1.48) in comparison of APAA, AT, NRT, and SAA when it compared with AA. In addition, there was no significant difference in the increase of short-term abstinence rates when comparing AT, NRT, or SAT with APAA, showing 0.65 (95% CI 0.29, 1.46), 0.65 (95% CI 0.30, 1.38), or 0.48 (95% CI 0.18, 1.18), respectively. There was also no significant difference in short-term abstinence rates between NRT and AT 0.99 (95% CI 0.56, 1.72) or SAT and AT 0.73 (95% CI 0.39, 1.25). The results of the network meta-analysis are displayed in Table 2.

#### 3.5.2. Long-Term Abstinence Rates

Data on long-term abstinence rates were available from 8 RCTs, reporting a total of 696 participants with events. The results of the pairwise random effect meta-analyses showed that RRs were low and without a significant difference, showing that AA versus SAT was 2.57 (95% CI 0.78, 8.63), APAA versus SAT was 1.96 (95% CI 0.59, 6.63), NRT versus SAT was 1.80 (95% CI 0.61, 5.51), AT versus SAT was 1.44 (95% CI 0.54, 3.90), and SAA versus SAT was 1.03 (95% CI 0.23, 4.29) (Figure 3). Network meta-analysis showed that only AA versus SAA had a statistical difference in long-term abstinence rate and RR value was 2.49 (95% CI 1.14, 5.97), but not among other interventions. The results of the network meta-analysis are shown in Table 2.

#### 3.5.3. FTND

Data on FTND scores were obtained from 6 RCTs; the direct meta-analysis showed that there was no significant difference in comparison between SAT and AA, APAA, AT, NRT, or SAA, showing that WMDs were −1.11 (95% CI −3.89, 4.20), 1.22 (95% CI −2.61, 5.23), 1.00 (95% CI −2.0, 3.99), 0.907 (95% CI −3.12, 4.90), or −0.324 (95% CI −4.49, 4.22) (Figure 3), respectively. Network meta-analysis showed that APAA versus AA was involved in two studies, and RR was 1.34 (95% CI −0.87, 3.16). Three studies involved SAA versus AA; RR was −0.19 (95% CI −1.9, 1.35). Comparisons between other interventions involved only one study, and none of the differences were statistically significant (Table 3).

#### 3.5.4. Daily Smoking

Seven studies reported changes in daily smoking. A total of four interventions were involved, including APAA, AA, SAA, and SAT. No significant difference was observed among these four groups (Figure 3). In addition, network meta-analysis showed that there was no statistical difference between the four interventions. Among them, AA versus APAA RR was −6.8 (95% CI −16.01, 2.39), APAA versus SAA RR was 7.69 (95% CI −2.36, 17.66), and AA versus SAT RR was −11.5 (95% CI −24.23, 1.24) (Table 3).

### 3.6. Adverse Events

Of the 23 studies we included, 17 of them show [21–25, 28–33, 35, 37–41] no information on adverse events. Two studies report no adverse events [11, 34]. One reports adverse events such as minor bleeding, hematoma, dizziness, fainting, residual needle sensation, tenderness, and minor infection during treatment (AT: 41 subjects; SAT: 44 subjects) [36]. Another study reports that 1 person in the AA group and 4 people in the SAA group have local ear maladjustment and 1 person in the SAA group had dizziness [26]. Two other studies report adverse reactions such as local ear maladaptation and pain during treatment [13, 27].

### 3.7. Inconsistency Analyses

Because only the network graph with short-term abstinence rates became a closed loop, we only performed node-splitting analysis, and node-splitting analysis showed no inconsistency between APAA and AA (*P*=0.75), AT and AA (*P*=0.74), NRT and AA (*P*=0.17), AT and APAA (*P*=0.18), NRT and AT (*P*=0.74), and SAT and AT (*P*=0.92). The results of direct comparison and indirect comparison were consistent (Supplementary Table 3).

### 3.8. Rank Probability

Table 4 shows the ranking of the six interventions; the smaller the SUCRA, the better. Both AA and APAA had the best effects in terms of short-term and long-term abstinence rates, and AA also ranked high in terms of daily smoking. APAA and AT had good effects on FTND scores. The SAT had the worst effect on short-term abstinence rates. SAA was the least effective for long-term abstinence rates and FTND scores.

### 3.9. Sensitivity Analysis

We used short-term abstinence rates, long-term abstinence rates, and daily smoking as indicators of sensitivity analysis. The clinical similarity and methodological similarity of the included studies were good, and the analytical results were reliable. Thus, our data were robust and reliable.

### 3.10. Publication Bias

**Figure 4:**
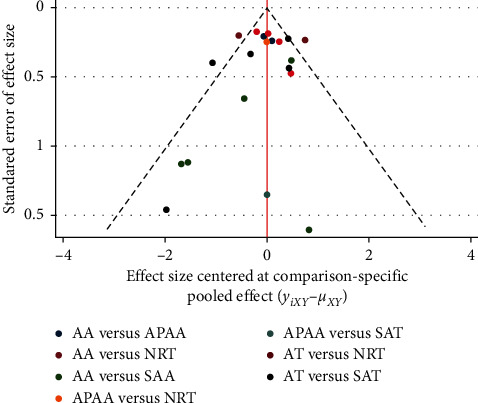
Funnel plot of short-term abstinence rates.

A funnel plot was performed for short-term abstinence rates, involving 18 studies, including a four arms study. It was divided into three separate studies. Funnel plots illustrated that scatters were almost visually symmetrical, and the result of Begg's test was *P*=0.82, meaning that publication bias was relatively low (Figure 4).

## 4. Discussion

It is well accepted that tobacco smoking is one of the most challenge public health problems nowadays, which also attracts enormous attention from medical and social points of view [42]. Quitting smoking probably is an effective way to reduce such damage [43]. Bayesian network meta-analysis was used for comparing the relative safety and efficacy of traditional Chinese medicine such as AT, AA, APAA, and NRT in smoking cessation. Although there was no significant difference among these four groups, using network meta-analysis, our data confirmed that the effects of smoking quit interventions among these four are similar, providing some useful information for smoking quitting. However, AA was superior to SAA in long-term abstinence rate comparison. The probability rankings of AA and APAA were also better than other interventions.

In addition to other sources of bias, most studies are not clear about whether the implementation of allocation concealment and blind method is correct, and the criteria are not clear, which may compromise the validity of the results. Particularly in the Chinese studies, most of them are subjective, which is more susceptible to affect the experimental outcomes objectively. We also consider that, due to the particularity of acupuncture and auricular acupressure, it may be difficult to treat blindness for doctors, but it is necessary to blind patients [26]. However, more than half of the studies have not performed blindness in this respect.

Most of the results of the Bayesian network meta-analysis were not statistically different, but the rank probability showed that AA and APAA were superior to other intervention indicators. It is worth noting that our estimates of the efficacy of these interventions are not accurate, which can be seen from the broad confidence interval. Moreover, the number of the included studies was relatively small. Some pairwise comparisons only involve one study. Therefore, more trials with a larger sample size are needed to establish more accurate efficient comparison. On the plus side, our paired meta-analysis results were consistent with the network meta-analysis results, which also improved the quality of our evidence. However, since most treatments do not have data on adverse events, the extent of their differences in safety is uncertain.

AT was not superior to other treatments such as SAT and NRT in the long-term abstinence rates, but there was a difference between AA and SAA, which was consistent with the previous research results. However, in terms of short-term abstinence rate, we did not conclude that AT or AA was superior to other therapies, which was consistent with the results of White et al., but different from those of Wang et al. [8, 44]. This may be related to the different interventions we have included. We excluded electroacupuncture and laser acupuncture only by incorporating pure acupuncture. Compared with previous studies, we may greatly reduce the heterogeneity between studies due to differences in intervention measures [8, 45]. Our research conclusions are not limited to the traditional acupuncture theory and are applicable to any therapy such as simple acupuncture and auricular acupressure. To our knowledge, there is no reticular network meta-analysis of acupuncture and auricular acupressure tablets for smoking cessation. The results of probability ranking showed that AA and APAA were superior to other interventions in smoking cessation. Compared with other interventions, AA is more convenient and safer. Based on the existing knowledge of auricular point sticking for smoking cessation, we can strengthen the application of such intervention, which will benefit more smokers, especially for those who have strong desire to quit smoking but have no free time, which may be a more practical and applicable intervention method. Therefore, we suggest that RCTs with standardized intervention measures, long intervention duration, high quality, and large sample size should be carried out in future clinical trial studies to clarify the advantages of AA. In addition, our results are based on random evidence, and we also summarize the safety profile of interventions such as acupuncture when quitting, which makes our research more comprehensive than the previous ones to provide a reference for clinical decision-making.

There were some limitations to our network meta-analysis. First, the course of treatment in the RCTs we included varied from 10 days to 8 weeks and the follow-up time was also quite different. Thus, it would be best to determine the collected data from a similar period. However, it would reduce the power of significance if a specific treatment period was selected in the current study. We will collect the data from larger data based which probably will enable us to test a more specific period. If the later clinical study can unify the treatment cycle and follow-up time, it may have been more convincing. Second, although the selected points of the studies we included are almost the same, there are also subtle differences. Although existing studies have shown that there is no direct linkage between acupoint selection and curative effect [14], acupuncture is a complex process, and the difference in the needling manipulation may also lead to different research results [46]. Therefore, uniform acupuncture manipulation is also an important point in the design of RCTs on smoking cessation by acupuncture in the future. This issue may be solved by comparing different interventions with more repeated RCTs in the future. Finally, because of the small number of trials reporting valuable measures such as Minnesota Nicotine Withdrawal Scale (MNWS) [47], Beck Anxiety Inventory (BAI) [48], and Beck Depression Inventory (BDI) [49], there was no correlation between the intervention groups, which would affect the judgment of potential efficacy, so we did not analyze these indicators.

Our analysis shows that a larger sample size, more consistent intervention methods, and treatment follow-up time are needed for future RCTs of acupuncture, auricular acupressure, and other traditional Chinese medicine methods for smoking cessation, so as to improve the quality of research and clarify the advantages of such methods in smoking cessation effect. In addition, we should pay more attention to the possible adverse events of these intervention processes and strengthen the evidence-based medical evaluation of safety evaluation. However, there is a lack of sufficient data from the included studies in the current network meta-analysis to offer an objectively dose-dependent value. This will be performed in our future study.

## 5. Conclusions

AA was superior to SAA in long-term abstinence rate comparison. The probability rankings of AA and APAA were also better than other interventions. RCTs with larger sample size, more consistent intervention methods, and longer treatment follow-up time will be investigated in future to further clarify the advantages of treatment methods such as APAA and AA. In addition, more attention should also be paid to the possible adverse events during the treatment of TCM methods such as acupuncture, which should be reported to supplement the evidence-based medical evidence in this area.

## Figures and Tables

**Table 1:** The characteristics of 23 included trials.

Study	Interventions	*N*	Proportion of females (%)	Mean age (year) (experimental group versus control group)	Dropout *n* (%)	Duration of treatment (T) and follow-up (F)	Outcomes
MacHovec et al., 1978	AT versus SAT	24	0	37	Not reported	T: 1 month F: 6 months	Abstinence rate (short-term)
Lamontagne et al., 1980	AT versus SAT	65	Not reported	33.2 ± 7.9/35.1 ± 6.8	Not reported	T: 1 month F: 6 months	Abstinence rate (short-term and long-term)
Zhang et al., 2004	AT versus SAT	60	0	25.70 ± 4.40/25.43 ± 3.66	Not reported	T: 4 weeks F: 1 year	Abstinence rate (short-term)
Wu et al., 2007	AT versus SAT	118	15.25	54.3 ± 16.9/53.0 ± 16.9	AT:5 (8.47) SAT:8 (13.56)	T: 8 weeks F: 6 months	Abstinence rate (short-term), mean daily smoking amount, FTND score
Hyun et al., 2010	AT versus SAT	80	6.25	40.0/42.0	AT:16 (42.1) SAT:18 (42.86)	T: 2 weeks F: 2 weeks	MNWS score, BDI score, BAI score
Chae et al., 2011	AT versus SAT	29	0	24.9 ± 2.6/25.0 ± 3.4	Not reported	T: 3 weeks F: 8 months	Exhaled CO level, HRV during visual cues, distraction to smoking cues, belief in and credibility of acupuncture, blinding indices
Kang et al., 2013	AT versus SAT	25	0	25.2 ± 2.0/24.8 ± 1.6	Not reported	Not reported	Exhaled CO level, craving scores to smoking-related visual cues
Ma et al., 2014	AT versus SAT	136	25.74	37–45	AT:12 (17.65) SAT:14 (20.59)	T: 8 weeks F: 6 months	Abstinence rate (short-term)
Wang et al., 2006	AA versus NRT	204	9.80	20–64	Not reported	T: 20 days F:/	Abstinence rate (short-term)
Li et al., 2009	AA versus SAA	140	23.57	38.23 ± 9.7/38.26 ± 9.56	AA:1 (1.43) SAA:3 (4.29)	T: 4 weeks F: 3 months	Abstinence rate (short-term)
Yeh et al., 2009	AA versus SAA	79	Not reported	28 ± 7.79/27 ± 7.63	AA:9(23.08) SAA:11(27.5)	T: 6 weeks F:/	Abstinence rate (short-term), serum cotinine levels, cigarette consumption, exhaled CO level
Wing et al., 2010	AA versus SAA	70	30.00	46.5 ± 12.36/46.4 ± 11.36	Not reported	T: 3 weeks F: 3 months	Abstinence rate (short-term), exhaled CO level, daily smoking amount
Ayse A-D, 2011	AA versus SAA	47	36.17	33.8 ± 10.26/34.0 ± 10.43	Not reported	T: 1 month F: 6 months	Abstinence rate (short-term and long-term), exhaled CO level, FTND score, BDI score
Zhang et al., 2013	AA versus SAA	43	58.14	50.4 ± 11.49/49.8 ± 8.53	Not reported	T: 8 weeks F: 3 months	Abstinence rate (short-term), cigarette consumption, daily smoking amount
Silva Rd.e et al., 2014	AA versus SAA	30	66.67	41	Not reported	T: 5 weeks F: 1 month	Exhaled CO level, FTND score, daily smoking amount
Lee et al., 2016	AA versus SAA	60	0	22.67 ± 2.02/22.19 ± 2.02	AA:3 (10.00) SAA:4 (13.33)	T: 6 weeks F:/	FTND score, exhaled CO level, self-efficacy for smoking cessation
Han, 2006	APAA versus AA	42	40.48	Not reported	Not reported	T: 10 days F: 1 month	Abstinence rate (short-term)
Liu et al., 2015	APAA versus AA	48	20.83	42 ± 8/40 ± 6	Not reported	T: 4 weeks F:/	Abstinence rate (short-term), daily smoking amount, FTND score, score of the tobacco dependence self-rating scale
Steiner et al., 1982	APAA versus SAT	32	34.38	Not reported	APAA:5 (31.25) SAT:4 (25.00)	T: 2 weeks F:/	Abstinence rate (short-term), group mean change in daily cigarette consumption, Tehchi sensations reported by treatment group
Clavel F et al., 1985	AT versus NRT	429	Not reported	Not reported	Not reported	T: 1 month F: 13 months	Abstinence rate (short-term and long-term)
Clavel F et al., 1997	AT versus NRT	485	55	All 34	Not reported	*T*: 6 months F: 4 years	Abstinence rate (short-term and long-term), exhaled CO level, FTND score, BDI score
Zhang et al., 2017	AT versus NRT	60	Not reported	Not reported	Not reported	T: 30 days F:/	Abstinence rate (short-term)
Chai et al., 2019	AT versus AA versus APAA versus NRT	400	6.5	43 ± 14/45 ± 14/46 ± 13/45 ± 14	Not reported	T: 8 weeks F: 16 weeks	Abstinence rate (short-term and long-term), FTND score, HIS score

**Table 2:** Network meta-analysis of the results on short-term abstinence rates and long-term abstinence rates (RR (95% CI)).

Short-term abstinence rates						
	AA	APAA	AT	NRT	SAA	SAT
AA	AA	1.01 (0.54, 1.9)	0.66 (0.31, 1.42)	0.65 (0.33, 1.3)	0.66 (0.3, 1.48)	0.48 (0.18, 1.17)
APAA	1.32 (0.62, 2.8)	APAA	0.65 (0.29, 1.46)	0.65 (0.3, 1.38)	0.65 (0.24, 1.82)	0.48 (0.18, 1.18)
AT	1.78 (0.89, 3.65)	1.36 (0.66, 2.82)	AT	0.99 (0.56, 1.72)	1 (0.34, 3.04)	0.73 (0.39, 1.25)
NRT	1.43 (0.7, 2.85)	1.09 (0.53, 2.2)	0.8 (0.48, 1.28)	NRT	1.01 (0.36, 2.96)	0.74 (0.32, 1.58)
SAA	2.49(1.14, 5.97)	1.89 (0.65, 6)	1.4 (0.49, 4.24)	1.75 (0.62, 5.38)	SAA	0.73 (0.2, 2.34)
SAT	2.57 (0.78, 8.63)	1.96 (0.59, 6.63)	1.44 (0.54, 3.9)	1.8 (0.61, 5.51)	1.03 (0.23, 4.29)	SAT
Long-term abstinence rates						

**Table 3:** Network meta-analysis of the results on FTND scores and daily smoking (WMD (95% CI)).

FTND scores						
	AA	APAA	AT	NRT	SAA	SAT
AA	AA	1.34 (−0.87,3.16)	1.13 (−1.88,3.53)	1.03 (−1.98,3.44)	−0.19 (−1.91,1.35)	0.11 (−4.2,3.89)
APAA	−6.8 (−16.01, 2.39)	APAA	−0.19 (−2.84, 2.19)	−0.3 (−2.94, 2.08)	−1.55 (−4.01, 1.15)	−1.22 (−5.23, 2.61)
AT	—	—	AT	−0.1 (−2.75, 2.56)	−1.35 (−4.25, 2.05)	−1 (−3.99, 2)
NRT	—	—	—	NRT	−1.25 (−4.17, 2.16)	−0.91 (−4.9, 3.12)
SAA	0.88 (−3.16, 4.84)	7.69 (−2.36, 17.66)	—	—	SAA	0.32 (−4.22, 4.49)
SAT	−11.5 (−24.23, 1.24)	−4.68 (−13.52, 4.17)	—	—	−12.38 (−25.7, 1.03)	SAT
Daily smoking						

**Table 4:** Rank probability of SUCRA.

Treatment	Short-term abstinence rates		Long-term abstinence rates		FTND scores		Daily smoking	
	SUCRA	Rank	SUCRA	Rank	SUCRA	Rank	SUCRA	Rank
AA	0.1848	1	0.0785	1	0.6802	5	0.7339	3
APAA	0.1945	2	0.3181	2	0.245	1	0.3337	2
AT	0.5715	3	0.6256	4	0.328	2	---	---
NRT	0.6033	5	0.3829	3	0.3727	3	---	---
SAA	0.5892	4	0.7994	6	0.7444	6	0.8702	4
SAT	0.8567	6	0.7956	5	0.6296	4	0.0622	1

## Data Availability

All data analyzed during this study are included in this published article and the original study publications.

## Supplementary Materials

Supplementary Materials Supplementary Table 1: search strategies. Supplementary Table 2: results of heterogeneity analysis. Supplementary Table 3: inconsistency analyses. Supplementary Figure 1: risk of bias summary. Supplementary Figure 2: risk of bias graph.
