# Supplementary material for: Comparison between Acupuncture and Nicotine Replacement Therapies for Smoking Cessation Based on Randomized Controlled Trials: A Systematic Review and Bayesian Network Meta-Analysis
Source: Evid Based Complement Alternat Med. 2021 Jun 16;2021:9997516. doi: 10.1155/2021/9997516 (PMC8225439; doi:10.1155/2021/9997516)
Supplement: Supplementary Materials — Supplementary Table 1: search strategies. Supplementary Table 2: results of heterogeneity analysis. Supplementary Table 3: inconsistency analyses. Supplementary Figure 1: risk of bias summary. Supplementary Figure 2: risk of bias graph. [file 9997516.f1.zip › 9997516.f1/Supplementary figure 1-Risk of bias summary.pdf]

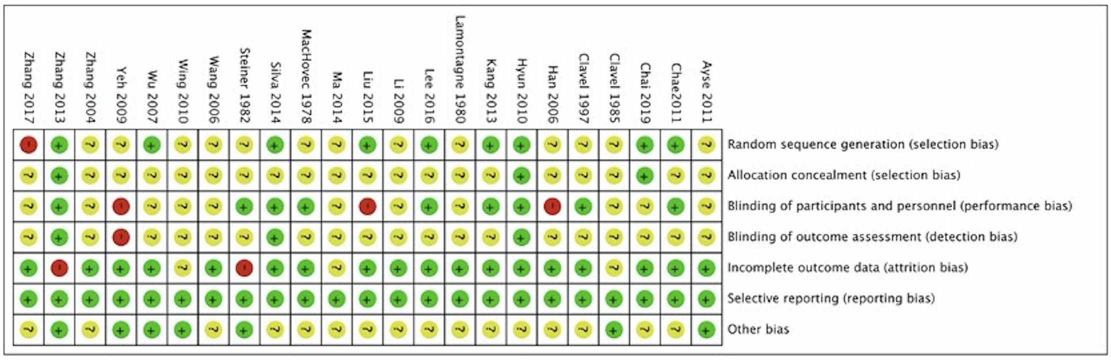
Zhang 2017
Zhang 2013
Zhang 2004
Yeh 2009
Wu 2007
Wing 2010
Wang 2006
Steiner 1982
Silva 2014
MacHovec 1978
Ma 2014
Liu 2015
Li 2009
Lee 2016
Lamontagne 1980
Kang 2013
Hyun 2010
Han 2006
Clavel 1997
Clavel 1985
Chai 2019
Chae2011
Ayse 2011
Random sequence generation (selection bias)
Allocation concealment (selection bias)
Blinding of participants and personnel (performance bias)
Blinding of outcome assessment (detection bias)
Incomplete outcome data (attrition bias)
Selective reporting (reporting bias)
Other bias
