# Supplementary material for: Comparison between Acupuncture and Nicotine Replacement Therapies for Smoking Cessation Based on Randomized Controlled Trials: A Systematic Review and Bayesian Network Meta-Analysis
Source: Evid Based Complement Alternat Med. 2021 Jun 16;2021:9997516. doi: 10.1155/2021/9997516 (PMC8225439; doi:10.1155/2021/9997516)
Supplement: Supplementary Materials — Supplementary Table 1: search strategies. Supplementary Table 2: results of heterogeneity analysis. Supplementary Table 3: inconsistency analyses. Supplementary Figure 1: risk of bias summary. Supplementary Figure 2: risk of bias graph. [file 9997516.f1.zip › 9997516.f1/Supplementary table 1-Search strategies (2).docx]

**Supplementary table 1：Search strategies**

| 1. Pubmed |
| --- |
| #1 Search "Smoking Cessation"[Mesh] |
| #2 Search ((Smoking*[Title/Abstract]) OR tobacco*[Title/Abstract]) |
| #3 Search #1 OR #2 |
| #4 Search "Nicotine Chewing Gum"[Mesh] OR "Tobacco Use Cessation Devices" [Mesh] |
| #5 Search ((Nicotine*[Title/Abstract]) OR Tobacco Use Cessation Devices [Title/Abstract]) |
| #6 Search #4 OR #5 |
| #7 Search " Auricular acupressure "[Mesh] |
| #8 Search Auricular acupressure* [Title/Abstract] |
| #9 Search #7 OR #8 |
| #10 Search "Acupuncture Therapy"[Mesh] |
| #11 Search ((((((Acupuncture*[Title/Abstract]) OR Pharmacoacupuncture* [Title/Abstract]) OR Acupotom*[Title/Abstract]) |
| #12 Search #10 OR #11 |
| #13 Search #6 OR #9 OR #12 |
| #14 Search #3 AND #13 |
| #15 Search #14: Randomized Controlled Trial |
| 1. Embase |
| #1 'smoking cessation'/exp |
| #2 ' nicotine*':ab,ti OR 'smoking*':ab,ti OR 'tobacco*':ab,ti |
| #3 #1 OR #2 |
| #4 'acupuncture'/exp |
| #5 'acupuncture*':ab,ti OR auriculotherapy:ab,ti |
| #6 #4 OR #5 |
| #7 'auricular acupressure'/exp |
| #8 'auricular acupressure':ab,ti |
| #9 #7 OR #8 |
| #10 'nicotine replacement therapy'/exp |
| #11 'nicotine replacement therapy':ab,ti |
| #12 #10 OR #11 |
| #13 #6 OR #9 OR #12 |
| #14 #3 AND #13 |
| #15 #14 AND 'randomized controlled trial'/de |
| 1. The Cochrane Library |
| #1 MeSH descriptor: [Smoking Cessation] explode all trees |
| #2 (Smoking*):ti,ab,kw OR (tobacco*):ti,ab,kw |
| #3 #1 OR #2 |
| #4 MeSH descriptor: [Acupuncture] explode all trees |
| #5(Acupuncture*):ti,ab,kw OR (Pharmacoacupuncture Therapy):ti,ab,kw OR (Acupotom*):ti,ab,kw |
| #6 #4 OR #5 |
| #7 MeSH descriptor: [Auricular acupressure] explode all trees |
| #8 (Auricular acupressure):ti,ab,kw |
| #9 #7 OR #8 |
| #10 MeSH descriptor: [Nicotine replacement] explode all trees |
| #11 (Nicotine*):ti,ab,kw OR (Smoking Cessation Product*):ti,ab,kw |
| #12 #10 OR #11 |
| #13 #6 OR #9 OR #12 |
| #14 #3 AND #13 |
| 1. Web of Science |
| #1 TS= (Smoking Cessations OR Stopping Smoking OR Giving Up Smoking OR Quitting Smoking OR Smoking Cessation) |
| #2 TS= (Acupuncture Treatment OR Acupuncture Treatments OR Pharmacoacupuncture Treatment OR Pharmacoacupuncture Therapy OR Acupotom* OR Acupuncture Therapy) |
| #3 TS= (Auricular acupressure) |
| #4 TS= (Nicotine * OR Smoking Cessation Product*) |
| #5 #2 OR #3 OR #4 |
| #6 #1AND#5 |
| #7 TS= ("randomized controlled trial" OR "multicenter study" OR "clinical trial" OR "single blind procedure" OR "controlled clinical trial" OR "double blind procedure") |
| #8 #6 AND #7 |
| 1. Chinese Biomedical Literature Database (CBM) |
| #1 "戒烟"[不加权:扩展] |
| #2 戒烟 |
| #3 #1OR#2 |
| #4 "针刺"[不加权:扩展] |
| #5 针刺 |
| #6 #4OR#5 |
| #7 "尼古丁替代"[不加权:扩展] |
| #8 尼古丁替代 |
| #9 #7 OR #8 |
| #10 "耳穴贴压"[不加权:扩展] OR "耳穴贴压, 王不留行"[不加权:扩展] |
| #11 耳穴贴压 |
| #12 #10 OR #11 |
| #13 #6 OR #9 OR #12 |
| #14 #3 AND #13 |
| #15 ((#13) AND (#3)) AND ("随机对照试验"[文献类型]) |
