# Supplementary material for: Comparison between Acupuncture and Nicotine Replacement Therapies for Smoking Cessation Based on Randomized Controlled Trials: A Systematic Review and Bayesian Network Meta-Analysis
Source: Evid Based Complement Alternat Med. 2021 Jun 16;2021:9997516. doi: 10.1155/2021/9997516 (PMC8225439; doi:10.1155/2021/9997516)
Supplement: Supplementary Materials — Supplementary Table 1: search strategies. Supplementary Table 2: results of heterogeneity analysis. Supplementary Table 3: inconsistency analyses. Supplementary Figure 1: risk of bias summary. Supplementary Figure 2: risk of bias graph. [file 9997516.f1.zip › 9997516.f1/Supplementary table 2-Results of heterogeneity analysis (2).docx]

**Supplementary table 2: Results of heterogeneity analysis**

| Treatment | Short-term abstinence rates | | | Long-term abstinence rates | | | FTND scores | | | Daily smoking | | |
| --- | --- | --- | --- | --- | --- | --- | --- | --- | --- | --- | --- | --- |
|  | *P* | *I^2^*(%) | *N* | *P* | *I^2^*(%) | *N* | *P* | *I^2^*(%) | *N* | *P* | *I^2^*(%) | *N* |
| AA vs APAA | NA | 72.84 | 3 | NA | NA | 1 | NA | 77.80 | 2 | NA | NA | 1 |
| AA vs AT | 0.75 | 0.00 | 1 | NA | NA | 1 | NA | NA | 1 | --- | --- | --- |
| AA vs NRT | 0.08 | 78.38 | 2 | NA | NA | 1 | NA | NA | 1 | --- | --- | --- |
| AA vs SAA | NA | 35.70 | 3 | NA | 0 | 3 | NA | 78.90 | 3 | NA | 71.9 | 5 |
| APAA vs AT | 0.25 | 71.91 | 1 | NA | NA | 1 | NA | NA | 3 | --- | --- | --- |
| APAA vs NRT | 0.36 | 55.29 | 1 | NA | NA | 1 | NA | NA | 3 | --- | --- | --- |
| APAA vs SAT | 0.92 | 0.00 | 1 | --- | --- | --- | --- | --- | --- | NA | NA | 1 |
| AT vs NRT | NA | 49.46 | 4 | NA | 0 | 3 | NA | NA | 1 | --- | --- | --- |
| AT vs SAT | NA | 75.15 | 5 | NA | 0 | 2 | NA | NA | 1 | --- | --- | --- |
