# Supplementary material for: Comparison between Acupuncture and Nicotine Replacement Therapies for Smoking Cessation Based on Randomized Controlled Trials: A Systematic Review and Bayesian Network Meta-Analysis
Source: Evid Based Complement Alternat Med. 2021 Jun 16;2021:9997516. doi: 10.1155/2021/9997516 (PMC8225439; doi:10.1155/2021/9997516)
Supplement: Supplementary Materials — Supplementary Table 1: search strategies. Supplementary Table 2: results of heterogeneity analysis. Supplementary Table 3: inconsistency analyses. Supplementary Figure 1: risk of bias summary. Supplementary Figure 2: risk of bias graph. [file 9997516.f1.zip › 9997516.f1/Supplementary table 3- Inconsistency analyses (2).docx]

**Supplementary table 3: Inconsistency analyses**

| Interventions | P | Direct | Indirect | Network |
| --- | --- | --- | --- | --- |
| AA.APAA | 0.7495200 | 0.087 (-0.53, 0.74) | -0.42 (-3.4, 3.3) | 0.0067 (-0.61, 0.63) |
| AA.AT | 0.7438467 | -0.30 (-1.6, 0.96) | -0.54 (-1.7, 0.67) | -0.41 (-1.2, 0.35) |
| AA.NRT | 0.1711933 | -0.64 (-1.4, 0.13) | 0.42 (-1.1, 1.8) | -0.42 (-1.1, 0.26) |
| APAA.AT | 0.1804600 | 0.0093 (-1.1, 1.1) | -1.0 (-2.1, 0.23) | -0.42 (-1.2, 0.38) |
| APAA.NRT | 0.2610467 | -0.067 (-1.2, 1.1) | -0.88 (-2.0, 0.24) | -0.43 (-1.2, 0.33) |
| APAA.SAT | 0.9323333 | -0.86 (-4.4, 1.8) | -0.74 (-1.9, 0.27) | -0.73 (-1.7, 0.16) |
| AT.NRT | 0.7435400 | 0.061 (-0.53, 0.61) | -0.47 (-3.5, 3.2) | -0.010 (-0.59, 0.54) |
| AT.SAT | 0.9212133 | -0.32 (-0.99, 0.25) | -0.48 (-4.1, 2.4) | -0.31 (-0.96, 0.22) |
