# Supplementary figures and images for: Comparison between Acupuncture and Nicotine Replacement Therapies for Smoking Cessation Based on Randomized Controlled Trials: A Systematic Review and Bayesian Network Meta-Analysis
Source: Evid Based Complement Alternat Med. 2021 Jun 16;2021:9997516. doi: 10.1155/2021/9997516 (PMC8225439; doi:10.1155/2021/9997516)

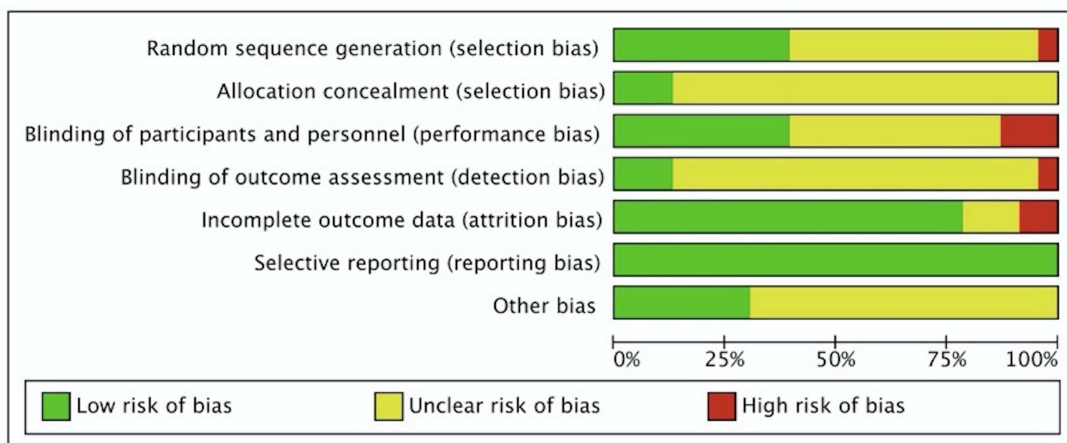

Supplement: Supplementary Materials — Supplementary Table 1: search strategies. Supplementary Table 2: results of heterogeneity analysis. Supplementary Table 3: inconsistency analyses. Supplementary Figure 1: risk of bias summary. Supplementary Figure 2: risk of bias graph. [file 9997516.f1.zip › 9997516.f1/Supplementary figure 2-Risk of bias graph.pdf]
